# Hope, agency, and adolescents' sexual and reproductive health: A mini review

**DOI:** 10.3389/frph.2023.1007005

**Published:** 2023-02-17

**Authors:** Candice Groenewald, Nazeema Isaacs, Phiwokazi Qoza

**Affiliations:** ^1^ Centre for Community-Based Research, Human Sciences Research Council, Pretoria, South Africa; ^2^ Psychology Department, Rhodes University, Grahamstown, South Africa; ^3^ Impact Centre, Human Sciences Research Council, Cape Town, South Africa

**Keywords:** adolescent & youth, agency, autonomy, sexual and reproductive health, self esteem, self-efficacay

## Abstract

Availability of and access to services that promote sexual and reproductive health (SRH) amongst adolescent girls have become a global priority. Yet, while researchers have explored factors that influence the uptake of SRH services in low-and-middle income countries, the roles that “agency” and “hope” play in adolescent SRH is less understood. To study this, this mini review systematically reviewed the literature across three databases, *EBSCO-host web*, *Pubmed* and *South Africa (SA) epublications*, for the period of January 2012 to January 2022. Findings showed that a paucity of studies identified the link between agency, hope and adolescent SRH respectively. Our review included 12 articles and found no studies that focused on hope and its role in adolescent SRH or seeking SRH services. However, the literature revealed the complexities of adolescent SRH agency and autonomy where female adolescents had limited autonomy to make SRH decisions. Limited access to adolescent friendly SRH services was also found to restrict girls' agency to prevent unintended pregnancies or to take up SRH support. Given the paucity of research, empirical studies are needed to further understand the extent to which hope, agency and other subjective factors implicate adolescent SRH in the African context.

## Introduction

Over the past two decades, there has been growing interest in the sexual and reproductive health (SRH) of adolescent girls and young women (AGYW) who have been identified as a particularly vulnerable group (1, 2). Adolescents are young people between the ages of 10 and 19 who experience socio-developmental transformations as they transition into adulthood (3, 4). These transformations are often associated with decision-making, establishing a social identity and self-concept, forming interpersonal relationships and physical changes in the body through puberty (4). Adolescent SRH relates to the freedom and rights that young people have to live healthy lives that are characterised by access to information, services, programmes and support that prevent, interrupt and/or address individual, social and systemic practices that put adolescents at risk of sexual violence, unwanted pregnancies, sexually transmitted infections and/or unsafe abortions (5). It also prioritises freedom of choice in accessing these services (6) and, in this way, challenges broader socio-cultural, religious and systemic practices that restrict access to SRH information and services.

While there have been remarkable improvements in the promotion of SRH knowledge and access to SRH services, globally, these gains have been inequitable in low-and-middle-income countries where the access, availability and quality of services have been inadequate (6). This is particularly evident in African countries where AGYW experience poverty and limited resources, gender inequality, gender-based violence and genital mutilation which significantly hinder access to and uptake of SRH services (7). Recent literature further indicates that adolescents in Sub-Saharan Africa (SSA) endure the highest burden of adverse SRH outcomes, where adolescent girls account for the largest number of new HIV-infections in East and SSA (8). More alarming is that women make up 72% of all young persons living with HIV in SSA while the global proportion of this cohort is lower at about 60% (9). It is therefore imperative to understand the factors that influence SRH amongst AGYW in African communities, to ensure that they have access to the highest attainable standard of health (10) and have knowledge and awareness of SRH information and their rights and agency to make informed SRH decisions.

## Agency, hope and adolescent SRH

This paper is concerned with the roles that agency and hope plays in the SRH of adolescents including accessing SRH services. Current SRH studies and programs have largely been directed towards enhancing AGYW's SRH knowledge and access to quality services, with a growing need to explore informed decision-making around their SRH (9). Agency refers to “the power to originate action” (11) and is often described as an ability that develops as we reflexively engage with social systems (12). According to Banerjee et al. (13), agency entails the ability to make strategic life choices through personal competence and self-efficacy. It implies a belief that the individual has the potential to direct a sequence of events that make the sum of lived experience. Pertaining to SRH, Vanwesenbeeck, and colleagues (14) uses the term sexual agency which describes young women's ability to make sexual choices, including initiating sex. Banerjee et al. (13) argues that a lack of SRH knowledge and information, and limited agency to negotiate sexual encounters contribute to early and unprotected sex for youth. Adolescents require agency to make health-related decisions, to access health services and to decide whether and when to engage in sexual relations and contraception (13). Social norms and community support enhance adolescents' agency and empower adolescent girls to make decisions in favour of their health such as postponing early marriage and their first childbirth as well as accessing SRH information and services (15). However, in SSA, young adolescents may have a general awareness about sexual and reproductive health matters, but lack in-depth knowledge to sufficiently protect themselves from sexual risks (16). Thus, Vanwesenbeeck et al. argue that agency is multifaceted and that the constructions of SRH agency need to consider the various contextual factors that limit adolescents decision-making (14).

Closely linked to agency, increasing attention has been given to the extent to which having hope shapes adolescent wellbeing (17–19). Hope is defined as an individual's “perceived capability to derive pathways to desired goals and motivate oneself via agency thinking to use those pathways” (18). Hope is the driver of agency, implicating the possibility to act necessary for the self-actualization of a goal. Studies have associated higher levels of hope with more positive outcomes in young people, including enhanced self-esteem, self-worth and self-care agency (19, 20). Hope has also been linked to decision-making and risk behaviours where adolescents have described young people who misused substances as having “no hope” (21) or associated hopelessness with engagement in adverse behaviours (17). Furthermore, hopelessness and other mental health indicators have been related to SRH amongst adolescents. A recent systematic review on the intersection between mental health and SRH amongst AGYW (22) shows that feelings of hopelessness, depression and diminished self-esteem have a bi-directional influence on young women's SRH. For example, unexpected pregnancies were associated with increased distress, hopelessness and depression while teenage pregnancy was identified as an emotional burden and linked to poor mental health (22). Diminished self-esteem was related to a lack of supportive environments and also described by young women as an outcome of controlling and violent sexual relationships (22).

While studies have examined the roles of hope and/or agency in adolescent health behaviour and outcomes, little is known about how these constructs intersect with adolescents' SRH practices in the African context. To study this, we conducted a systematic search of the literature, using set criteria, to identify published studies conducted in Africa on these topics. This mini review thus synthesises the identified literature to explore how these subjective factors influence adolescents' SRH and access to SRH services.

## Review approach

This mini-review consulted three databases, namely, EBSCO-host web, SA epublications and Pubmed, making use of Boolean phrase options within databases. The first set of keywords used were “agency” OR “autonomy” OR “self determination” OR “self esteem” OR “self concept” AND “adolescents” OR “teenagers” OR “teen” OR “youth” AND “sexual reproductive health” OR “sexual reproductive health services” AND “Africa”. Apart from the term “Africa”, only articles with the respective keywords in the abstracts were deemed relevant. This strict criterion was applied to ensure that we identify articles that directly link to our interest area and, in this way, avoid articles that merely mention the respective keywords in the body of the text. This search produced the following, EBSCO-host web (*n* = 455), SA epublications (*n* = 25), and Pubmed (*n* = 36). The second keyword search were “hope” OR “hopefulness” OR “self efficacy” AND “adolescents” OR “teenagers” OR “teen” OR “youth” AND “sexual reproductive health” OR “sexual reproductive health services” AND “Africa”. This search produced the following, EBSCO-host web (*n* = 18), SA epublications (*n* = 8), and Pubmed (*n* = 124).

**Figure 1:**
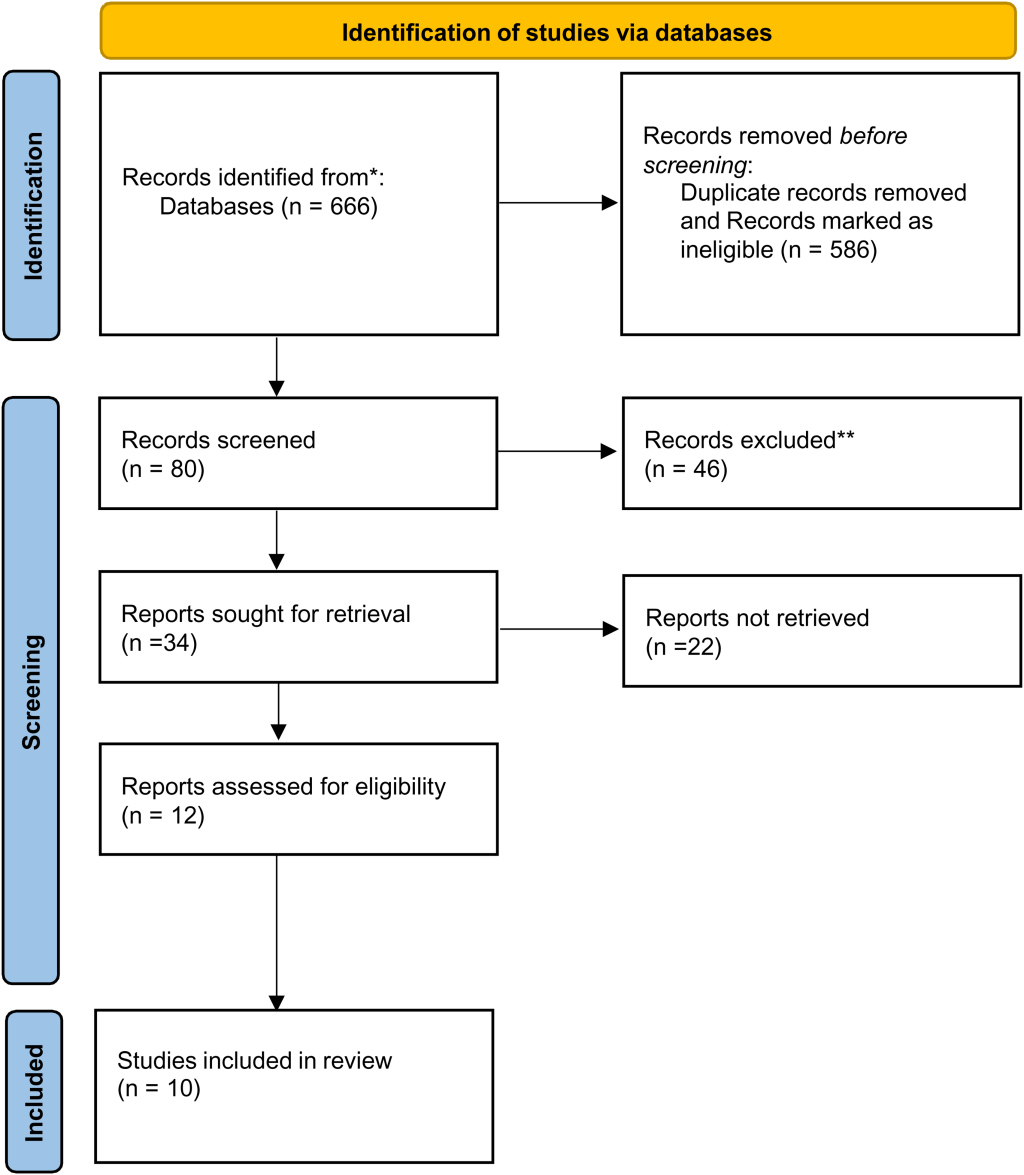
Prisma diagram to screen articles. Page MJ, McKenzie JE, Bossuyt PM, Boutron I, Hoffmann TC, Mulrow CD, et al. The PRISMA 2020 statement: an updated guideline for reporting systematic reviews. BMJ 2021;372:n71. doi: 10.1136/bmj.n71.

Following this initial search, we conducted an abstract level assessment based on the inclusion criteria such as the timeline (i.e., January 2012 to June 2022), only peer-reviewed articles, English language, and studies including empirical data. The three reviewers used these criteria, together with the primary objective that only articles that relate to the link between hope and SRH (including services) or agency and SRH (including services), to assess the relevance of each abstract and whether it was included or excluded. After the abstract assessment, 34 articles were selected for full text assessment. Full text articles were independently assessed by three authors for methodological rigor and to avoid bias (see Figure 1). The final review included a total of (*n* = 10) articles (See Appendix A).

Furthermore, we also conducted quality appraisals of the articles included in this paper using two frameworks. For papers that employed qualitative methods (including the mixed methods study), we used the Long and colleagues' revised critical appraisal skills programme (CASP) tool (23), while we appraised quantitative papers using the Study Quality Assessment Tools proposed by the National Institute of Health (NIH) (24).

## Results

### Descriptive profile of papers

Most of the papers were published in 2021 (*n* = 7), followed by 2020 (*n* = 1), 2019 (*n* = 1) and 2016 (*n* = 1). Although most papers were recently published, no studies focused directly on the COVID-19 pandemic. As shown in Tables 1, 2, the majority of the articles made use of quantitative approaches (*n* = 5) (29–33), followed by qualitative- (*n* = 4) (25–27, 34), and one mixed methods study (*n* = 1) (28). Of the studies using quantitative methods, one (*n* = 1) used a randomized evaluation design, three (*n* = 3) used a randomized control trial (including the mixed methods study) and two (*n* = 2) used a cross-sectional design. In terms of context, the articles focused on Kenya (*n* = 1), Ghana (*n* = 1), South Africa (*n* = 4), Uganda (*n* = 1), Zambia (*n* = 2), and Liberia (*n* = 1).

**Table 1:** Study population clearly described including participation criteria. Power calculations provided.

Included article	Thematic area	Methodology		Was the research question or objective in this paper clearly stated?	Was the study population clearly specified and defined?	Was the participation rate of eligible persons at least 50%?	Were all the subjects selected or recruited from the same or similar populations (including the same time period)? Were inclusion and exclusion criteria for being in the study prespecified and applied uniformly to all participants?	Was a sample size justification, power description, or variance and effect estimates provided?	For the analyses in this paper, were the exposure (s) of interest measured prior to the outcome (s) being measured?	Was the timeframe sufficient so that one could reasonably expect to see an association between exposure and outcome if it existed?	For exposures that can vary in amount or level, did the study examine different levels of the exposure as related to the outcome (e.g., categories of exposure, or exposure measured as continuous variable)?	Were the exposure measures (independent variables) clearly defined, valid, reliable, and implemented consistently across all study participants?	Was the exposure (s) assessed more than once over time?	Were the outcome measures (dependent variables) clearly defined, valid, reliable, and implemented consistently across all study participants?	Were the outcome assessors blinded to the exposure status of participants?	Was loss to follow-up after baseline 20% or less?	Were key potential confounding variables measured and adjusted statistically for their impact on the relationship between exposure (s) and outcome (s)?
Pöllänen K, de Vries H, Mathews C, Schneider F, de Vries, PJ. Beliefs About Sexual Intimate Partner Violence Perpetration Among Adolescents in South Africa. *Journal of Interpersonal Violence.* (2021) 36 (3–4): NP2056–2078NP	Self-efficacy	*Cross-sectional study, performing secondary data analyses on baseline data from 42 randomly selected high schools with young people aged 12–23. The number of adolescents who participated were 3,251, although only 2,199 formed part of the analysis in the paper. This is based on the inclusion criteria that only participants who indicated having had a boyfriend or girlfriend were included.	**Review criteria**	Aim of the paper and relevance of study clearly described	Study population clearly described including participation criteria. Power calculations not provided	55% of those invited to partake in the study, participated.	Yes, all participants recruited from public high schools using the same inclusion/exclusion criteria	Sample size justification provided- including sample size justification of secondary analysis sample	No, this paper uses a cross-sectional study design.	No, this is a cross-sectional study	Yes for some variables.	Yes	N/A	Researchers used objective measures that were previous used in similar studies to measure outcome variable. However, the question was one that asked participants to self-report behaviour without an additional objective measure [which would be difficult to do]. All measures were consistenly implemented across study participants	N/A this is a cross-sectional study	N/A this is a cross-sectional study	Yes, regression models were used
Austrian K, Kangwana B, Muthengi1 E, Soler-Hampejsek, E. Effects of sanitary pad distribution and reproductive health education on upper primary school attendance and reproductive health knowledge and attitudes in Kenya: a cluster randomized controlled trial. *Reproductive Health*. (2021) 18:179: 1–13.	Self-esteem	* Longitudinal, cluster-randomized controlled trial in 140 public primary schools accross three rural sub-counties. The study enrolled 3,489 randomly selected girls in primary school grade 7 with a mean age of 14 years.		Aim of the paper and relevance of study clearly described	Study population clearly described including participation criteria. Power calculations provided	Yes	Yes, all participants recruited from schools using the same inclusion/exclusion criteria	Yes	Yes	Yes, approximately 20 months	Yes for some variables.	Yes	Yes	Researchers used objective measures but did no indication of whether these are standardised tests, previously used or self-developed. All measures were consistenly implemented across study participants	Yes	Yes	Yes, regression models were used
Lolla D, Fleminga PJ, Stephensonb R, Kinga EJ, Morhec E, Manud A, Halle KS. Factors associated with reproductive autonomy in Ghana. *Culture, Health & Sexuality.* (2021) 23 (3): 349–366.	Autonomy	Cross-sectional study using secondary data from a community-based survey focused on sexual and reproductive related stigma. The sample consisted of 516 young women between the ages of 15–25.		Aim of the paper and relevance of study clearly described	Study population clearly described including participation criteria. Power calculations not provided	Not described	No, samples recruited from different spaces. Eligibility based on age, location and language and appears to have been applied uniformly across spaces	Sample size justification provided- including sample size justification of secondary analysis sample. No power description	No, this paper uses a cross-sectional study design.	No, this is a cross-sectional study	Yes for some variables.	Yes	N/A	Researchers used objective measures that were previous used in similar studies to measure outcome variable. All measures were consistenly implemented across study participants	N/A this is a cross-sectional study	N/A this is a cross-sectional study	Yes, regression models were used
Kemigisha E, Bruce K, Ivanova O, Leye E, Coene G, Ruzaaza1 GN, Ninsiima AB, Mlahagwa W, Nyakato VN, Michielsen K. Evaluation of a school based comprehensive sexuality education program among very young adolescents in rural Uganda. *BMC Public Health.* (2019) 19 (1393): 1–11	Self-esteem	* A mixed methods approach was used. The cluster randomized trial among 1,096 pupils in 33 randomly selected primary schools. This was followed by a qualitative evaluation of an intervention in 4 schools that consisted of 14 in-depth interviews and 3 focus group discussions.		Aim of the paper and relevance of study clearly described	Study population clearly described including participation criteria. Power calculations provided	Yes	Yes, all participants recruited from local schools using the same inclusion/exclusion criteria	Yes	Yes	Yes approximately 1 year	Yes for some variables.	Yes	Yes	Researchers used objective measures that were previous used in similar studies to measure outcome variable. All measures were consistenly implemented across study participants	No	Yes with caution at 20,6%	Yes, regression models were used
Firestone R, Moorsmith R, James S, Urey M, Greiffnger R, Lloyd D, Hartenberger-Toby L, Gausman J, Sanoeg M. Intensive Group Learning and On-Site Services to Improve Sexual and Reproductive Health Among Young Adults in Liberia: A Randomized Evaluation of Healthy Actions. *Global Health: Science and Practice.* (2016) 4(3): 435–451	Self-esteem	Randomized evaluation design with young women and men ages 15–35. 1,157 learners were assessed at baseline and 1,052 learners were assessed at endline		Aim of the paper and relevance of study clearly described	Study population clearly described including participation criteria. Power calculations provided	Yes	Yes, all participants recruited from similar populations and same inclusion criteria were used. However a new sample was selected at a later period due to loss to follow up.	Yes	Yes	Yes, endline conducted after exposure to all intervention content concluded	Unclear	Yes	Yes	Researchers used objective measures but did no indication of whether these are standardised tests, previously used or self-developed. All measures were consistenly implemented across study participants	No	Yes	Yes, regression models were used
Austrian K, Soler-Hampejsek E, Behrman JR, Digitale J, Hachonda JN, Bweupe M, Hewett PC. The impact of the Adolescent Girls Empowerment Program (AGEP) on short and long term social, economic, education and fertility outcomes: a cluster randomized controlled trial in Zambia. *BMC Public Health.* (2020) 20 (349):1–15.	Self-efficacy	*A cluster-randomized-controlled trial with longitudinal observations. Baseline data were collected from never-married adolescent girls in 120 intervention clusters (3,515 girls) and 40 control clusters (1,146 girls) and again two and four years later.		Aim of the paper and relevance of study clearly described	Study population clearly described including participation criteria. Power calculations provided	Unclear	Yes, all participants recruited from similar populations and same inclusion criteria were used.	Yes	Yes	Yes, multiple follow ups over 4 years	Yes for some variables.	Yes	Yes	Researchers used objective measures that were previous used in similar studies to measure outcome variable. All measures were consistenly implemented across study participants	No	Yes	Yes, regression models were used

**Table 2:** Aim of the paper and relevance of study clearly described.

Included article	Thematic area	Methodology		Clear statement of study aims	Appropriateness of qualitative method	Appropriateness of design to address aims	Theoretical underpinnings of study clearly stated	Appropriateness of recruitment strategies	Appropriateness of data collection methods	Researcher reflexivity	Ethical issues outlined	Rigour of data analysis	Clear statement of findings	Value of research clearly articulated
Rousseau E, Bekker L, Julies RF, Celum C, Morton J, Johnson R, Baeten JM, O’Malley G. A community-based mobile clinic model delivering PrEP for HIV prevention to adolescent girls and young women in Cape Town, South Africa. *BMC Health Services Research*. (2021) 21(888):1–10 (25)	Self-efficacy	Qualitative interviews with 30 (N) participants between the ages of 16–25	**Review criteria**	Aim of the paper and relevance of study clearly described	Not well articulated or justified but meets the goal of the study	Design appropriate to meet study aims, but justification for design not well articulated	Not mentioned	Recruitment strategy described in detail, however no information provided on participants who refused to participate. Almost 40 percent of the participants who were approached refused to participate.	Data collection methods clearly described but not well justified. Setting for data collection was well justified along ith data collection approaches. Sample questions from interview guide provided and references are made to how data was captured (audio), transcribed and that saturation was achieved.	Not mentioned	Ethics approval number provided but no details on different ethical considerations applied in the study	Authors described the coding process conducted by the study team. However, no mention of a particular analyses methdology. Authors make use of representative extracts in paper, and where appropriate offer comparisons of participants experiences or compare participants narraitves to contextual expectations	Findings clearly articulated in discussion and linked to broader literature. Authors also criticlaly engage with their finidngs in relation to contextual and structural factors and offer recommendations. Data presented as preliminary and limitations to generalisability are noted.	Authors discuss the relevance of study to broader audiences and offer recommendations for research and intervention
Adams L, Crowley T. Adolescent human immunodeficiency virus self-management: Needs of adolescents in the Eastern Cape. Afr J Prm Health Care Fam Med. (2021)13 (1): a2756. https://doi.org/10.4102/phcfm.v13i1.2756 (26)	Self-esteem and self-efficacy	Qualitative interviews with 13 (N) participants between the age of 14 and 19 years were interviewed.		Aim of the paper and relevance of study clearly described	Not well articulated or justified but meets the goal of the study	Design appropriate to meet study aims, but justification for design not well articulated	Theoretical framework offered	Recruitment strategy described in detail and reasons provided on why some participants were excluded or did not participate.	Data collection methods clearly described but not well justified. Setting for data collection was well justified. Sample questions from interview guide provided and references are made to how data was captured (audio), transcribed and that saturation was achieved.	Author describes position and relationship to study sites (independent) and provides some thoughts on how researcher position influenced the study	Ethics approval number provided with details on different ethical considerations applied in the study	Authors describe the coding process conducted by the study team. Author also identifies an analyses methdology. Authors make use of representative extracts in paper, and where appropriate offer comparisons of participants experiences or compare participants narraitves to contextual expectations	Findings clearly articulated in discussion and linked to broader literature. Authors also criticlaly engage with their findings in relation to contextual and structural factors and offer recommendations. Limitations to the study are described in terms of the sample group and potential bias due to language used during interviews	Authors discuss the relevance of study to broader audiences and offer recommendations for intervention
Munakampe MN, Michelo C, Zulu JM. A critical discourse analysis of adolescent fertility in Zambia: a postcolonial perspective. *Reproductive Health*. (2021). 18(75): 1–12 (27)	Agency and autonomy	Qualitative data collection with adolescents aged 15–19, including 25 individual interviews and 9 focus group discussions (*N* = 67).		Aim of the paper and relevance of study clearly described	Qualitative method justified and meets goal of the study	Design appropriate to meet study aims	Not mentioned	Recruitment strategy described in detail, however no information provided on participants who refused to participate. Participants selected by community health care workers or teachers, but criteria not outlined.	Data collection methods described but not well justified. Setting for data collection was well justified along. Sample questions from interview guide provided and references are made to how data was captured (audio)and that it was transcribed	Not mentioned	Ethics approval number provided with details on different ethical considerations applied in the study	Authors describe the analytic process and frameworks that guided the analyses. Authors make use of representative extracts in paper, and where appropriate offer comparisons of participants experiences or compare participants narraitves to contextual expectations and to other sample groups in the study	Findings clearly articulated in discussion and linked to broader literature. Authors also criticlaly engage with their finidngs in relation to contextual and structural factors and offer recommendations. Limitations to generalisability are noted.	Authors discuss the relevance of study to broader audiences and offer implications for research and practice.
Gillespie B, Allen H, Pritchard M, Soma-Pillay *P*, Balen J, Anumba D. Agency under constraint: Adolescent accounts of pregnancy and motherhood in informal settlements in South Africa. *Global Public Health.* (2021):1–13. Doi: 10.1080/17441692.2021.1981974 (25)	Agency	Qualitative interviews with 40 (N) participants between the ages of 16 to 32.		Aim of the paper and relevance of study clearly described	Not well articulated or justified but meets the goal of the study—no reflection on positionality.	Design appropriate to meet study aims, but justification for design not well articulated	Conceptual framework offered	Recruitment strategy not well described however study context described. No information provided on participants who may have refused to participate	Data collection methods described but not well justified. Setting for data collection also described. No sample questions provided and references are made to how data was captured (fieldnotes only)	Not mentioned	Ethics approval number provided with details on different ethical considerations applied in the study	Authors describe the coding process conducted by the study team. Author also identifies an analyses methdology but does not go into detail. While authors make use of representative extracts in paper, very few are used. Where appropriate, comparisons of participants narratives are offered	Findings clearly articulated in discussion and linked to broader literature. Authors also criticlaly engage with their finidngs in relation to contextual and structural factors and offer recommendations. Limitations to generalisability are noted.	Authors discuss the relevance of study to broader audiences and offer implications for research and practice. Recommendations not explicitly articulated
**Kemigisha E, Bruce K, Ivanova O, Leye E, Coene G, Ruzaaza1 GN, Ninsiima AB, Mlahagwa W, Nyakato VN, Michielsen K. Evaluation of a school based comprehensive sexuality education program among very young adolescents in rural Uganda. *BMC Public Health.* (2019) 19(1393): 1–11 (28)	Self-esteem	Mixed methods study including an initial cluster randomized trial with pupils in 33 randomly selected primary schools, followed by a qualitative evaluation of the intervention in 4 schools. Qualitative component entailed 4 interviews with pupils along with 2 focus groups (*N* = 16). Interviews and focus group discussions were also conducted with parents and teachers		Aim of the paper and relevance of study clearly described	Qualitative method justified and meets goal of the study	Design appropriate to meet study aims	Conceptual framework offered in relation to intervention design	Recruitment strategy for qualitative component not well described however study context described in relation to evaluation. No information provided on participants who may have refused to participate	Data collection methods described but not well justified. Setting for data collection also described. No sample questions provided and references are made to how data was captured (audio) and that it was transcribed	Not mentioned	Ethics approval number provided with details on different ethical considerations applied in the study	Authors described the coding process conducted by the study team. However, no mention of a particular analyses methdology. Authors make use of representative extracts in paper, and where appropriate offer comparisons of participants experiences or narraitves	Findings clearly articulated in discussion and linked to the quantitative findings and broader literature. Authors also criticlaly engage with their finidngs in relation to contextual and structural factors and offer recommendations. Limitations to generalisability pertaining to the qualitative component are noted.	Authors discuss the relevance of study to broader audiences and offer implications for research, policy and practice.

### Agency, hope and sexual and reproductive health

Of the articles that were relevant, studies focused on self-efficacy and self- esteem (*n* = 2), and agency and autonomy (*n* = 3). Authors also evaluated programmes that generally aimed to enhance uptake of SRH services amongst adolescents (*n* = 5). No studies in our sample examined the association between hope and SRH amongst adolescents as framed by our inclusion criteria.

## Self-Efficacy and self-esteem

Self-efficacy and self-esteem were identified as important factors in adolescents SRH practices. Adams & Crowly (26) examined the self-management practices and needs of older adolescents (14–19 years) living with HIV in a community in South Africa. They found that amongst those participants who were sexually active, some felt pressured to have sex without a condom and had lower self-efficacy to negotiate condom use with their partners as they had not disclosed their HIV status to their partners (26). Non-disclosure was also associated with enhanced self- esteem and confidence because the secrecy of their HIV status protected the participants from experiencing stigma or othering in their social environments (26). Further, non-disclosure, or keeping their HIV status confidential, played a part in how treatment taking was managed where participants would make concrete efforts (like going somewhere private or taking medication before socialising) to hide their status (26). It was, however, unclear whether this influenced treatment adherence or whether self-esteem or self-efficacy influenced accessing SRH services. Yet, findings did show that some participants did not consider interactions with their healthcare workers as important and felt demotivated to communicate with them due to “the attitude of certain healthcare workers, a large number of patients at the clinics and long waiting times” (26).

The link between self-efficacy and SRH behaviours was also examined by Pöllänen et al. (29) who investigated South African adolescent boys' and girls' experiences and intentions towards perpetrating sexual violence within their relationships. They found that boys not only had attitudes that were more supportive of committing sexual intimate partner violence than girls, but they also had “a lower self-efficacy to refrain from pressurizing a boyfriend or girlfriend to have sex than girls” (29). In the overall sample, self-efficacy to refrain from pressurizing a partner to have sex was also lower in those who had indicated that they had committed sexual intimate partner violence (29). Across both studies included here, self-efficacy influenced sexual behaviours either through an inability to negotiate condom use in sexual relationships (26) or an inability to resist pressurizing a partner to have sex (29).

## Agency and autonomy

The role of agency and autonomy in SRH practices and behaviours was discussed in three papers. Munakampe, Michelo and Zulu (27) explored the various discourses that influence adolescent fertility in Zambia. Their findings showed that female adolescents had limited autonomy, or “control of their fertility” which subsequently led to early marriage and early childbearing cultures within the country (27). And while early marriage and childbearing was associated with agency to access SRH services, adolescent girls' agency was compromised by diminished financial independence and reduced autonomy to use SRH information and services. Limited adolescent autonomy was also reinforced by elders in the community, such as parents, teachers or healthcare workers, which subsequently increased adolescents' acceptance of restricted autonomy to make SRH related decisions (27).

Restricted autonomy amongst adolescent females has also been reported in Ghana where Lolla, et al. (30) explored SRH decision-making and communication autonomy of young women. Focusing on females aged 15 to 24 years, Lolla et al. (30) found that older women, women who have never been pregnant, and who have increased social support for adolescent SRH were more comfortable to discuss (communication autonomy) SRH related issues (contraception, pregnancy etc.) with their male partners had more autonomy than young adolescents with no support. On the other hand, increased decision- making autonomy (having control over SRH decisions) was observed amongst younger participants and those who have never been pregnant (30). In this regard, the authors concluded that reproductive autonomy is complex where younger women are more comfortable to talk about reproductive decisions with their male partners but did not necessarily have the “the most say in [making] decisions” around their SRH (30).

Similar findings were also reported in South Africa by Gillespie, et al. (25) who explored adolescents' perspectives of falling pregnant and motherhood in informal settlements. Gillespie et al. (25) found that adolescents' agency to prevent unintended pregnancies was compromised by limited adolescent-friendly SRH services and a lack of communication on SRH practices including issues of safe sex and contraception. Some adolescents reported that they were not comfortable to access SRH services in their community due to fears of being judged and limited privacy at the clinics.

Adolescents thus had limited access to contraceptives and condoms and limited information on safe sex practices. Supporting Munakampe et al. (27) and Lolla et al. (30), the authors concluded that adolescents' agency is multifaceted and that constructions of SRH agency need to consider the various contextual factors that “limit their potential space for action and decision-making” while recognising the attempts that adolescents continue to make to manage their decisions.

## Services and programmes

Majority of the included studies evaluated SRH programmes (including services and education programs) for adolescents across different African contexts. In South Africa, the Tutu Teen Truck (TTT) mobile health clinic was developed to improve adolescents access to SRH services (34). Services provided through the TTT included HIV testing, STI testing and treatment, and a range of contraceptives (oral, injectable, and implant). Majority of the adolescent girls who participated in the study used contraceptives, started on PrEP and tested for STIs (34). The findings revealed that TTT made SRH services accessible to adolescents as it eliminated logistical and travel barriers and operated through short visits rather than lengthy appointments (34). In addition, AGYW indicated that positive provider-client interactions they fostered with the TTT strengthen self-efficacy (34). In Kenya, adolescents in primary schools were selected to form part of the NIA project intervention (31). One part of the intervention included adolescent girls receiving one packet of ten disposable sanitary pads monthly and two pairs of underwear, while the other part included a 25-session curriculum delivered by trained facilitators as part of extra-curricular activities in schools (31). The authors reported that although the intervention improved girls' attitudes and self-efficacy, increasing their pride and comfort, it did not improve adolescent girls' attendance in school during their menstruation period.

The remaining three papers reported on the value of education programs to enhance youth SRH knowledge and facilitate positive SRH behaviours. This included the evaluation of the HealthyActions curriculum in Liberia where out of school youth were provided with SRH information and access to products and services (32). An increase in the use of contraceptives and HIV testing centres (HTC) were found among the study sample at the end of the study. Positive SRH outcomes were also reported in Southwestern Uganda where researchers evaluated an intervention designed to enhance SRH knowledge and practices with primary school adolescents (28). Findings showed improved SRH knowledge and increased understandings of SRH related risks (28). Enhanced SRH knowledge was also identified as an outcome of the Adolescent Girls Empowerment Program (AGEP) which was implemented in ten sites across four provinces in Zambia (33). AGEP included three intervention components: firstly, weekly group meetings; secondly, provision of a health voucher to be used at contracted public and private facilities; and thirdly, provision of an adolescent-friendly savings account (33). Findings showed that there was a sustained change in SRH knowledge and self-efficacy, and savings, but the shorter-term changes did not result in longer-term impacts on education or fertility for vulnerable girls in the Zambian context (33). It is evident to state that the findings of the SRH programmes within the included articles revealed that there was improvement with adolescent's self-efficacy, however, it did not focus on agency or hope in relation to adolescents SRH in the African context.

## Quality appraisal

The final step in our review was to conduct a risk of bias assessment of the studies using two separate frameworks for papers employing qualitative methods and those using quantitative approaches, as outline previously. Summaries of these assessments are presented in Tables 1, 2. Using Long et al.' s framework (23), Table 1 shows that a primary area for concern amongst the qualitative studies was limited descriptions or justifications on the applicability of qualitative methods to meet the study goals. This limitation was observed in 3 of the 5 studies which may pose a risk of bias and draw caution to how the findings of these studies are interpreted. Other risks included inadequate justifications of the appropriateness of qualitative data collection approaches, where authors generally identified approaches (i.e., individual interviews or focus group discussions) without justification. Only one paper referred to the author's positionality within the research process but did not unpack how this potentially influenced the study outcomes. While all the qualitative papers used representative extracts to present the qualitative data, two papers did not identify a credible analytic technique and majority employed a group coding approach. This collective coding approach may enhance inter-rated credibility of the findings, but this was not unpacked in the studies. Further, an accepted limitation of qualitative studies is restrictions of generalizability of findings which most studies recognized. However, to address these issues, studies adequately related their findings to the broader literature, drawing, also, implications for research policy or practice.

The quantitative studies included in this review generally used structured assessment techniques. As indicated in Table 2, studies that employed evaluation designs provided power estimations on how the sample were identified which enhances the generalizability of the study findings to the broader population. Majority of the studies recruited participants from similar contexts (generally through schools) and all studies uniformly applied their inclusion/exclusion criteria during recruitment. Intervention studies conducted pre-tests and post-test to assess changes in outcomes due to intervention exposure and collected post-test data between 1 year and 4 years after conclusion of intervention. These studies also showed good retention rates where loss to follow-up after baseline was 20% or less. Papers generally described the assessment tools used in the studies, but some did not indicate whether these tools were self-developed or derived from established tools. This may present a risk of bias as it is difficult to assess the validity of such measures (24). However, all papers used methodologically sound analytic approaches and generally relied on regressions which are considered reliable approaches to take account for the influence of confounding variables (24).

In all, the quantitative studies offered less risks of bias, given the structured and objective approaches used to gather and analyze the data. The qualitative studies on the other hand, showed less room for such objectivity, which is not unexpected given the subjective nature of qualitative research. However, qualitative papers used established data collection approaches (although not always justified), and usually employed a group coding approach to assess the groundedness (strength) of codes and sub-themes. These approaches enhance the credibility of the findings which were also interpreted in relation to the literature.

## Discussion

The aim of this review was to explore the literature on hope and agency in adolescents' SRH in the African context. While research has explored the factors that influence uptake of SRH services in low-and-middle income countries, our findings show that the role of hope and agency in accessing SRH services is less understood. Importantly, no studies in our sample examined the association between hope and adolescent SRH, highlighting a significant gap in the literature. While we also included the keyword “hopefulness” as part of our Boolean search, this too generated no studies that were directly linked to adolescent SRH. Still, enhanced hope has been associated with decreased participation in health-related risky behaviours such as alcohol use (21) while hopelessness and poor mental health have been associated with an increased likelihood of risk behaviours such as perpetration of sexual violence (35) or other violence behaviours (36). Empirical research to study how hope influences adolescent SRH practices, behaviours and access to services in the African context is warranted.

Studies also explored the link between self-efficacy, self-esteem, agency and autonomy, and adolescents SRH, respectively. While the influence of these constructs in adolescents seeking of SRH services was not a primary focus in some studies, self-efficacy was found to influence sexual behaviours either through an inability to negotiate condom use in sexual relationships (26) or an inability to resist pressurizing a partner to have sex (29). The literature also revealed the complexities of adolescent SRH agency and autonomy where female adolescents had limited autonomy and experienced early marriage and childbearing (27) or did not have “the most say” in their own SRH decisions (30). Agency and autonomy were also linked to uptake of SRH services. Limited access to adolescent friendly SRH services restricted girls’ agency to prevent unintended pregnancies (27) or to take up SRH support (25).

Majority of the included articles discussed SRH programmes to improve adolescents access to SRH services. These included a mobile clinic to deliver SRH services (34), distribution of SRH products to sustain school involvement during menstruation (31), education programs (28, 32, 33). In general, these studies reported positive results where uptake of SRH services like PrEP, HIV testing, contraceptive use amongst adolescent girls, was found (32, 34). Education programs was also found to increase SRH knowledge (28, 31, 33). While the distribution of sanitary pads did not improve school attendance during menstruation, the program did enhance adolescent girls' self-efficacy and positive attitudes towards menstruation (31). While positive results were reported in these studies, further research is necessary to assess the feasibility of these interventions in different countries and communities.

Multi-country investment is pertinent to identify contextually relevant and culturally sensitive interventions and programmes to enhance adolescent SRH and prevent, interrupt, and address the various barriers to accessing adolescent friendly SRH services and products. Contextual factors not only play significant roles in shaping the quality, availability, and accessibility of SRH services and programs for AGYW, but also regulates how young people make decisions or enact their agency and constructions of hope (14, 17, 37). For example, studies show that AGYW's ability to exercise autonomy over their bodies is constrained by traditional, cultural, and religious norms that view publicly expressing sexuality and talking about sex as taboo (38–41). This was also reported in the literature that formed part of this mini review (27, 31–33). Consequentially, accessing sexual and reproductive health services are hindered by limited dialogues about sex and sexuality, or judgemental attitudes that AGYW receive from older healthcare workers (42–44). Young women also fear experiencing stigma and shame after clinic visits as some remain doubtful about health care worker confidentiality (45). Furthermore, the literature in this study also draws attention to impact that wider structural constraints, like poverty and living conditions can have on adolescents' agency and SRH. There was general recognition of the limited availability of SRH services in low resource communities where Rousseau and colleagues (34), for example, called for SRH services that use diverse modalities to deliver services such as the mobile health clinic.

## Conclusion

This mini review highlights the importance of understanding the role of individual factors like hope, self-efficacy, agency, and autonomy in adolescent SRH, behaviours and access to services. Studies are necessary to also consider how these factors interact to either facilitate or compromise safe SRH practices and access to services amongst adolescents. Recognising the influence of the social environment in examining these interactions is key, given the important roles that culture, religion and traditional norms play in shaping risk and protective SRH behaviours in African contexts (27, 38–41). Many AGYW are hindered from accessing SRH services due to experiences and fears of being condemned, feeling judged and not being able to engage in open dialogue about sex and sexuality with relatively older health care workers (25, 27, 42, 44–46). In this regard, we support Desmond et al. (21) who argues that we need “to understand how individuals and groups understand their environment, how they interact with and change their environment, and how these understandings and interactions shape behaviour [and access to support or intervention]”.

Moreover, this paper is not without limitations. The review included papers published within particular journals and databases, and within a set timeframe where we may have excluded other relevant. Importantly, access to full text articles were provided through our institutional subscriptions, which may have further restricted access to articles published in journals to which the institution does not subscribe. Our criteria also strictly included empirical studies while other systematic reviews (if available) or unpublished dissertations could offer additional insights into the relationships between hope or agency and adolescent SRH. Papers also focused only on studies that include adolescents within their samples and we therefore acknowledge that further understandings can be gleaned from the broader literature on adult youth SRH.

## Appendix A

a. Rousseau E, Bekker L, Julies RF, Celum C, Morton J, Johnson R, Baeten JM, O’Malley G. A community-based mobile clinic model delivering PrEP for HIV prevention to adolescent girls and young women in Cape Town, South Africa. *BMC Health Services Research*. (2021) 21(888):1–10
b. Pöllänen K, de Vries H, Mathews C, Schneider F, de Vries, PJ. Beliefs About Sexual Intimate Partner Violence Perpetration Among Adolescents in South Africa. *Journal of Interpersonal Violence.* (2021) 36(3–4): NP2056–2078NP
c. Austrian K, Kangwana B, Muthengi1 E, Soler-Hampejsek, E. Effects of sanitary pad distribution and reproductive health education on upper primary school attendance and reproductive health knowledge and attitudes in Kenya: a cluster randomized controlled trial. *Reproductive Health*. (2021) 18:179: 1–13.
d. Adams L, Crowley T. Adolescent human immunodeficiency virus self-management: Needs of adolescents in the Eastern Cape. *Afr J Prm Health Care Fam Med.* (2021)13(1): a2756. https://doi.org/10.4102/ phcfm.v13i1.2756
e. Munakampe MN, Michelo C, Zulu JM. A critical discourse analysis of adolescent fertility in Zambia: a postcolonial perspective. *Reproductive Health*. (2021). 18(75): 1–12
f. Lolla D, Fleminga PJ, Stephensonb R, Kinga EJ, Morhec E, Manud A, Halle KS. Factors associated with reproductive autonomy in Ghana. *Culture, Health & Sexuality.* (2021) 23(3): 349–366.
g. Gillespie B, Allen H, Pritchard M, Soma-Pillay P, Balen J, Anumba D. Agency under constraint: Adolescent accounts of pregnancy and motherhood in informal settlements in South Africa. *Global Public Health.* (2021):1–13. doi: 10.1080/17441692.2021.1981974
h. Kemigisha E, Bruce K, Ivanova O, Leye E, Coene G, Ruzaaza1 GN, Ninsiima AB, Mlahagwa W, Nyakato VN, Michielsen K. Evaluation of a school based comprehensive sexuality education program among very young adolescents in rural Uganda. *BMC Public Health.* (2019) 19(1393): 1–11
i. Firestone R, Moorsmith R, James S, Urey M, Greiffnger R, Lloyd D, Hartenberger-Toby L, Gausman J, Sanoeg M. Intensive Group Learning and On-Site Services to Improve Sexual and Reproductive Health Among Young Adults in Liberia: A Randomized Evaluation of Healthy Actions. *Global Health: Science and Practice.* (2016) 4(3): 435–451
j. Austrian K, Soler-Hampejsek E, Behrman JR, Digitale J, Hachonda NJ, Bweupe M, Hewett PC. The impact of the Adolescent Girls Empowerment Program (AGEP) on short and long
k. term social, economic, education and fertility outcomes: a cluster randomized controlled trial in Zambia. *BMC Public Health.* (2020) 20(349): 1–15.
